# Wound Botulism in Injection Drug Users

**DOI:** 10.3201/eid1306.061336

**Published:** 2007-06

**Authors:** Wiltrud Maria Kalka-Moll, Ute Aurbach, Reiner Schaumann, Rosemarie Schwarz, Harald Seifert

**Affiliations:** * University of Cologne Medical Center, Cologne, Germany; † University of Leipzig, Leipzig, Germany; ‡ Municipal Hospital of Cologne, Cologne, Germany

**Keywords:** wound botulism, heroin, abscess, Clostridium botulinum, antitoxin, PFGE, outbreak, letter

**To the Editor:** Infections are the most frequent and serious wound complications in injection drug users (IDUs). Wound botulism is primarily caused by *Clostridium botulinum* (*1*) and was first observed in IDUs in New York in 1982 (*2*). It results from the introduction of *C. botulinum* spores into a wound and their multiplication, germination, in situ synthesis, and secretion of toxin under anaerobic conditions. Of 7 designated toxin types, neurotoxins A, B, E, and F result in human disease. During the 1990s, wound botulism cases among IDUs increased in the United States in conjunction with the use of black-tar heroin (*3*). Since 2000, wound botulism cases in IDUs have been reported in Europe (*4*). To our knowledge, molecular epidemiologic analyses have not been performed to confirm suspected outbreaks.

Within a 6-week period in October and November 2005, 12 clinical cases were recognized in the metropolitan area of Cologne, Germany (*5*). Six patients were successfully treated at teaching hospitals of the University of Cologne. On admission, all socially nonrelated patients had signs of bilateral symmetric cranial neuropathies such as ptosis, diplopia, blurred vision, dysphagia, dysarthria associated with symmetrical descending weakness of the upper extremities, and no sensory deficiencies. Treatment of patients included administration of trivalent A, B, and E antitoxin; antimicrobial drugs such as penicillin G or mezlocillin with metronidazole; and surgical drainage of any existing abscesses.

Patient 1, a 31-year-old female IDU, had multiple abscesses on both legs. Four days after her admission, wound botulism was suspected and antitoxin administered. Respiratory failure required mechanical ventilation for 11 weeks. Patient 2, a 51-year-old male IDU, had 1 large abscess on the left lower leg. Antitoxin was administered within 3 days of hospital admission. Mechanical ventilation was required for 5 weeks. Patient 3, a 25-year-old male IDU, had a large abscess on the left forearm. Patient 4, a 43-year-old man who used heroin intramuscularly, had an abscess of moderate size on the left forearm. Antitoxin was administered within 12 hours of admission to patients 3 and 4, and both patients required 2 weeks of respiratory support. Patient 5, a 32-year-old male IDU who was positive for hepatitis C virus, had purchased heroin from the same dealer as patient 2. Abscesses were absent. Antitoxin was administered within several hours of admission. Within 10 days, the patient recovered fully without need for mechanical ventilation. Patient 6, a 44-year-old male IDU, had several skin lesions at injection sites on his arms, but no abscesses. He received antitoxin treatment within several hours of admission and was discharged with minimal residual neck weakness after 7 days.

Serum specimens were obtained from patients 1, 2, 5, and 6. Botulinum toxin detected by the mouse bioassay in serum of patients 1 and 2, but not of patients 5 and 6, was neutralized by polyvalent antitoxin (Novartis Behring, Marburg, Germany). Abscess specimens were available from patients 2, 3, and 4. Anaerobic cultures grew *C. botulinum,* which was identified by Gram stain, culture morphologic features, Rapid ID 32A (bioMérieux, Marcy l’Etoile, France), and 16S rDNA sequencing. All strains were susceptible to penicillin G and metronidazole, as determined by the E-test (AB Biodisk, Solna, Sweden). PCR assays performed for *C. botulinum* type A, B, E, and F neurotoxin genes (*6*,*7*) identified the single toxin B. Toxin B production was confirmed by the mouse bioassay. Pulsed-field gel electrophoresis (PFGE) after *Sma*I, *Sac*II, and *Xho*I restriction (*8*) showed indistinguishable strains from patients 2, 3, and 4 (shown for *Sma*I in the Figure).

**Figure:**
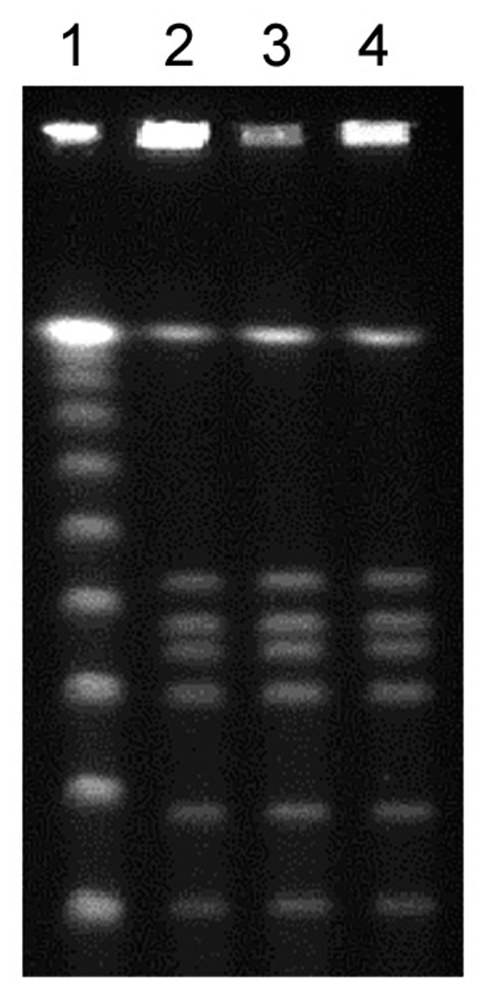
Fingerprint patterns obtained for *Clostridium botulinum* isolates following pulsed-field gel electrophoresis after *Sma*I restriction show identical strains. Lane 1, 100-bp ladder; lanes 2–4, abscess fluid isolates from patients 2, 3, and 4, respectively.

To our knowledge, this is the first outbreak of wound botulism in IDUs that was confirmed by molecular epidemiologic typing. PFGE suggests a single-source exposure with *C. botulinum* type B in at least 3 IDUs; this implies that the heroin was obtained from a common source, where contamination with *C. botulinum* spores may have been introduced when mixed with adulterants or diluted with substances such as dextrose or dyed paper. Skin popping (subcutaneous and intramuscular injection), which may increase the odds of wound botulism by a factor >15 (*9*), was used by all patients for drug delivery. This study confirms previous observations that the duration of clinical symptoms before antitoxin administration affects the need for and duration of mechanical ventilation (*10*). Here, the time from hospital admission to antitoxin treatment ranged from several hours to 4 days and correlated with the mechanical ventilation interval ranging from 0 days to 11 weeks. In addition, the extent of abscesses, which ranged from no abscesses to multiple abscesses, seems to affect clinical outcome. As soon as an index case of wound botulism in IDUs is diagnosed, a coordinated public health case-management effort, including hospitals, outpatient clinics, and information centers for drug addicts, is mandatory to alert the medical community and the drug users to consider wound botulism if typical symptoms occur and to enable the prompt administration of antitoxin. Obtaining tissue samples or abscess fluid for culture and molecular epidemiologic studies of *C. botulinum* isolates is necessary to facilitate identification of the source of the contaminated heroin.
